# Deep Learning Technology Applied to Medical Image Tissue Classification

**DOI:** 10.3390/diagnostics12102430

**Published:** 2022-10-07

**Authors:** Min-Jen Tsai, Yu-Han Tao

**Affiliations:** 1 Institute of Information Management, National Yang Ming Chiao Tung University, 1001 Ta-Hsueh Road, Hsinchu 300, Taiwan; 2 National Applied Research Laboratories, Taiwan Instrument Research Institute, 20, R&D Rd. VI, Hsinchu Science Park, Hsinchu 300, Taiwan

**Keywords:** convolutional neural network, deep learning, colorectal cancer classification

## Abstract

Medical image classification is a novel technology that presents a new challenge. It is essential that pathological images are automatically and correctly classified to enable doctors to provide precise treatment. Convolutional neural networks have demonstrated their effectiveness in classifying images in deep learning, which may have dozens or hundreds of layers, to illustrate the relationship between them in terms of their different neural network features. Convolutional layers consisting of small kernels take weights as input and guide them through an activation function as output. The main advantage of using convolutional neural networks (CNNs) instead of traditional neural networks is that they reduce the model parameters for greater accuracy. However, many studies have simply been focused on finding the best CNN model and classification results from a single medical image classification. Therefore, we applied a common deep learning network model in an attempt to identify the best model framework by training and validating different model parameters to classify medical images. After conducting experiments on six publicly available databases of pathological images, including colorectal cancer tissue, chest X-rays, common skin lesions, diabetic retinopathy, pediatric chest X-ray, and breast ultrasound image datasets, we were able to confirm that the recognition accuracy of the Inception V3 method was significantly better than that of other existing deep learning models.

## 1. Introduction

Artificial intelligence (AI) can revolutionize the diagnosis of diseases by performing classifications that are difficult for human experts and quickly providing large numbers of images. CNNs have been used to classify medical images [1] and detect cancerous tissue in histopathological images [2] from tissue microarrays (TMAs) of human tumors based on chromatin patterns [3], extract predictors [4], and classified tumor nuclei. The traditional classification approach involves manually segmenting images and then using a classifier or a classifier trained by shallow neural computers to identify each segmentation and classify the image [5]. However, methods for building and improving classifiers are time consuming and computationally expensive [6,7,8].

The development of convolutional neural networks in recent years has significantly improved the results of image classification [9,10]. Among them, transfer learning has proven to be an extremely effective technique, especially when there are limited data [11,12,13]. The main advantage of using CNNs instead of traditional neural networks is that they reduce the model parameters for greater accuracy. CNNs use a convolutional neural network with multiple processing layers to build the features of the images in each layer. After the feature map has been generated, it can be used as the input for the next layer. This architecture can process images in pixel form as input, and provide the desired classification as output. Transfer learning involves the use of data from similar domains to compensate for the lack of localized data. Weights in lower layers that have been optimized are used to identify the structure of general images and retrain weights in upper layers with backpropagation for the faster identification of category-specific images.

The improvement of neural networks is a common research goal in deep learning, and most studies are focused on data augmentation [14,15,16,17], comparison of model structures [18,19,20], or widely used regression loss functions [21,22]. This study aimed to compare six common deep neural networks based on CNN models to identify the best-performing deep learning technique for processing different medical images to accurately and opportunely diagnose key pathologies. After conducting experiments on six publicly available databases of pathological images, including colorectal cancer tissue, chest X-rays, common skin lesions, diabetic retinopathy, pediatric chest X-ray, and breast ultrasound image datasets. We further compared the adaptive moment estimation (Adam) optimizer and stochastic gradient descent momentum (SGDM) optimizer, replacement mini-batch size, and epoch to test the model. Therefore, this successfully demonstrates the wide application of the Inception V3 model in classifying different medical images. 

To achieve this goal, it is necessary to:

- Compare the classification accuracy rate of different CNN models.
- Find the best performing deep learning technique.
- Compare it with the results of existing techniques and methods.

Deep learning and its applications in classifing pathological images are systematically addressed in this paper. The relation to past studies of deep learning are reviewed in Section 2, while the approach of deep learning models is described in detail in Section 3. Section 4 contains the experiment and the paper is concluded in Section 5 with suggestions for a possible future investigation of this field.

## 2. Related Works

Most of the past studies that were based on deep learning models that could be used to automatically classify histopathological images had six deep learning network models in common, i.e., VGG19, AlexNet, GoogLeNet, SqueezeNet, ResNet50, and Inception V3. The current methods that are used to classify histopathological images are described below, and the accuracy of their results is shown in Table 1.

### 2.1. Predicting Colorectal Cancer Slice Categories

Kather et al. [23] used 100,000 histological colorectal cancer images (NCT-CRC-HE-100K) for model training, including nine tissue classes. They then split the image dataset into three data stores, trained 70%, and tested and validated 15% each, without any overlaps. After that, they used five different CNN models to train the dataset: VGG19 [25], AlexNet [9], GoogLeNet [24], SqueezeNet [26] and ResNet50 [27]. All models were pretrained on the ImageNet database, and the network was trained using stochastic gradient descent with SGDM after replacing the classification layers. The classification layer with the best accuracy rate of 98.7% was replaced with VGG19 [23].

Additionally, they used an external validation set of 7180 images (CRC-VAL-HE-7K) and achieved a best prediction classification accuracy of 94.3%. The main contribution of VGG19 is that the architecture is composed of two convolutional layers using the ReLU activation function, followed by a max-pooling layer. At the same time, the fully connected layer also uses the ReLU activation function, and the last layer is a Softmax layer for classification. VGG19 proved that the depth of the network was the key to achieving classification accuracy and that CNNs could directly evaluate and predict tumors from histopathological images.

### 2.2. Weakly Supervised Classification of Chest X-ray Diseases

As proposed by Wang [28], we used a framework composed of weakly supervised multi-label image classification and pathology localization to detect the presence or absence of one or more pathologies in X-ray images by training a multi-label DCNN classification model. The ChestX-ray8 database consisted of 108,948 frontal X-ray images, 24,636 of which contained one or more pathologies. The remaining 84,312 images were normal. We randomly split the entire dataset into 70% training, 10% validation, and 20% testing via stochastic gradient descent (SGD) for the pathology classification and localization tasks. ImageNet [29,30] was used as a pretrained model, and AlexNet, GoogLeNet, VGGNet-16, and ResNet-50 were used to omit the fully connected layer and the final classification layer. A transition layer, global-pooling layer, and prediction layer were inserted and a loss layer was placed at the end. Among these models, ResNet-50 achieved the best result of 69.67% as a unified framework for the assessment and validation of disease classification and localization in the ChestX-ray8 database. 

### 2.3. Different Dermoscopic Images

Past studies of the diagnosis of pigmented skin lesions based on using artificial neural networks were mainly focused on melanoma and moles due to the small range of training data, while diverse types of non-melanocytic lesions in real-life data were ignored. To facilitate research of the automatic diagnosis of dermoscopy images, Tschandl [31] released the HAM10000 (“Human Against Machine with 10,000 training images”) dataset. The Inception V3 [32] architecture is fine-tuned with weights pretrained on ImageNet4 data using hand-labeled images as the training dataset. After training with stochastic gradient descent for 20 epochs with a learning rate initialized to 0.0003 and a batch size of 64, the images were classified according to their type with 95% accuracy.

### 2.4. Retinopathy Identification

Daniel [33] constructed a deep learning-based framework and proposed the use of transfer learning to replace multiple steps of traditional methods. This architecture could process images in a pixel format as input and provide the desired classification as output. In this study, we took advantage of transfer learning due to limited data in order to train a neural network with a small subset of data to identify specific image features in retinal tomography OCT images. The model was trained using 108,312 OCT images from 4686 patients (37,206 choroidal neovascularization, 11,349 diabetic macular edema, 8617 drusen, and 51,140 normal). Among these, diabetic macular edema, which is a vision-threatening form of diabetic retinopathy, is likely to increase in prevalence over time due to an aging population and the global diabetes epidemic. The model was tested with 1000 images (250 per class) of 633 patients. An accuracy of 93.4% was achieved in a multiclass comparison between choroidal neovascularization, diabetic macular edema, drusen, and normal samples. A limited model was trained using 1000 images of 633 patients (classified between the same four classes, 250 images each) to compare transfer learning with limited data performance and large datasets, and 93.4% accuracy was achieved using the same test images.

### 2.5. Detection of Pneumonia

To determine if the deep learning framework of Daniel [33] could diagnose common diseases, the same transfer learning framework was applied to classify pediatric chest X-rays, detect pneumonia, and distinguish between viral and bacterial pneumonia to improve rapid referral. To train the model, 5232 pediatric chest X-ray images were collected from 5856 patients and labeled. A total of 3883 of these images were described as pneumonia (2538 bacteria and 1345 viruses) and 1349 were described as normal. The model was tested with 234 normal images and 390 pneumonia images (242 bacteria and 148 viruses) of 624 patients. An accuracy of 92.8% was achieved in experiments comparing chest radiographs showing pneumonia with normal chest radiographs.

### 2.6. Detection of Breast Ultrasound Images

Walid [33] found that a combination of breast ultrasound images and machine learning produced good results in terms of the classification, detection, and segmentation of breast cancer, but it also contained a great deal of unimportant information. For the dataset to be useful, the data needed to be preprocessed to remove duplicate images, which reduced the number of images to 780. Firstly, 1100 raw images were collected and stored in a DICOM format acquisition at Baheya Hospital, and the three common breast pathology classifications frequently observed and diagnosed were listed as normal, benign, and malignant. A folder was created for each category and images of each category were placed in the designated folders. The image name included the category name and the image number to construct and provide a public dataset of breast ultrasound images that are useful for deep learning in future research.

## 3. Research Method

Deep learning has become extremely popular as a means of solving complex problems. Six common deep neural network architectures based on CNN models [9,27,32,34,35,36,37,38] were used in this study to compare the accuracy of different medical image classifications.

### 3.1. Experimental Steps

The CNN model recognition process can be divided into three stages, the first of which is training the model, the second is collecting the necessary data, and the third is testing the model.

#### 3.1.1. Finding the Best Architecture

Six common deep neural networks based on CNN models were used in this study.

(1) AlexNet

AlexNet [9] is a widely applied deep convolutional neural network, which can still achieve a competitive performance in terms of classification when compared to other kinds of networks. In the training step of the AlexNet model, the input image is resized to 224 × 224 pixels and fed into the network. The architecture of AlexNet firstly adopts a convolutional layer to perform convolution and max pooling with local response normalization (LRN), in which 96 different receptive filters of size 11 × 11 are used. Its operational filters are performed in the second layer, and the max-pooling operations are performed with 3 × 3 filters. The third, fourth, and fifth convolutional layers use 384, 384, and 296 feature maps. The output of the two fully connected (FC) layers is used as an extracted feature vector, with dropout followed by a softmax layer at the end to classify problems.

(2) ResNet

The accuracy of this model does not improve as the number of network layers increases and a more complex feature extraction is performed because test and training errors will significantly improve after the network deepens. Therefore, deeper models do not necessarily achieve better results. ResNet [27] has a residual learning framework for ultra-deep networks, and the residual function can alleviate the networks that do not disappear due to the vanishing gradient problem. The main architecture of the ResNet network is one input layer, four convolutional layers, and one output layer. The difference between ResNet18 and ResNet50 networks is that there are many differences in the block parameters and the number of intermediate convolutional layers.

(3) Inception V3

Inception V3 [32] is mainly focused on reducing computing power consumption by modifying the previous Inception architecture. This idea was proposed in a paper in 2015 entitled “Rethinking the Inception Architecture for Computer Vision”. Several techniques for optimizing the network are proposed in the Inception V3 model, including decomposed convolutions, regularization, dimensionality reduction, and parallel computing. The 3 × 3 Convolution is decomposed into two one-dimensional convolution concatenations (1 × 3 and 3 × 1), which can accelerate the calculation further. It increases the network depth, thereby increasing the nonlinearity of the network (ReLU is required for each additional layer). Factorization, which decomposes the 7 × 7 convolution into two one-dimensional concatenations, is a major improvement in Inception V3.

(4) DenseNet

The DenseNet [36] model is a dense mechanism that connects the front and back layers, each of which accepts the previous layer as input, so that the input layer has more connections. Moreover, DenseNet directly connects the feature maps of different layers, and the reuse feature enables it to achieve a good performance with fewer parameters and computational costs. This feature is the main difference between DenseNet and ResNet.

(5) MobileNet

MobileNet [37] is a lightweight network architecture that replaces the standard convolutional layers in VGG. There are two main types of convolutions: spatially separable and depthwise separable. Spatially separable convolution turns one large convolution kernel into two small ones, while depthwise separable convolution is a kind of factorized convolution, which can be decomposed into two smaller operations: depthwise convolution and pointwise convolution. Separable convolution is applied in MobileNet V1, and two hyperparameters are proposed to control the network capacity. The assumptions behind this convolution are cross-channel and cross-spatial correlation. Depthwise separable convolution can save the number of citations and achieve fairly high accuracy while maintaining acceptable model complexity on the mobile side. MobileNet V2 applies a new unit: inverted residual with a linear bottleneck. The main features are the addition of a linear-enabled output to the bottleneck and the transfer of the skip-connection structure of the residual network to a low-dimensional bottleneck layer.

(6) XceptionNet

Xception [38], the extreme version of Inception, is another improvement to Inception V3 that was proposed by Google after Inception. Its linear stacking contains depthwise separable convolutional layers with residual connections. The main purpose of Xception is not to compress the model, but to improve its performance by mainly replacing the convolution operation in the original Inception V3 with a depthwise separable convolution. Xception widens the network so that the number of parameters is similar to Inception V3, thereby improving the model’s effect without increasing the complexity of the network.

#### 3.1.2. Data Availability

Firstly, we downloaded six different open histology image datasets and organized their image numbers (Table 2). In the experiments, we split different partitions for training, validation, and testing sets for fair comparison. Specifically, the training, validation, and test sets are defined as follows:

Training set: a training dataset is a dataset of examples used during the learning process and is used to fit the parameters (e.g., weights) of, for example, a classifier.

Validation set: a validation dataset is a dataset of examples used to tune the hyperparameters (i.e., the architecture) of a classifier. An example of a hyperparameter for artificial neural networks includes the number of hidden units in each layer.

Test set: a test dataset is a dataset that is independent of the training dataset, training, validation and test sets cannot have any overlap. In this way, testing can really measure the capabilities of the model.

(1) Colorectal Cancer Tissue

**Figure 1:**
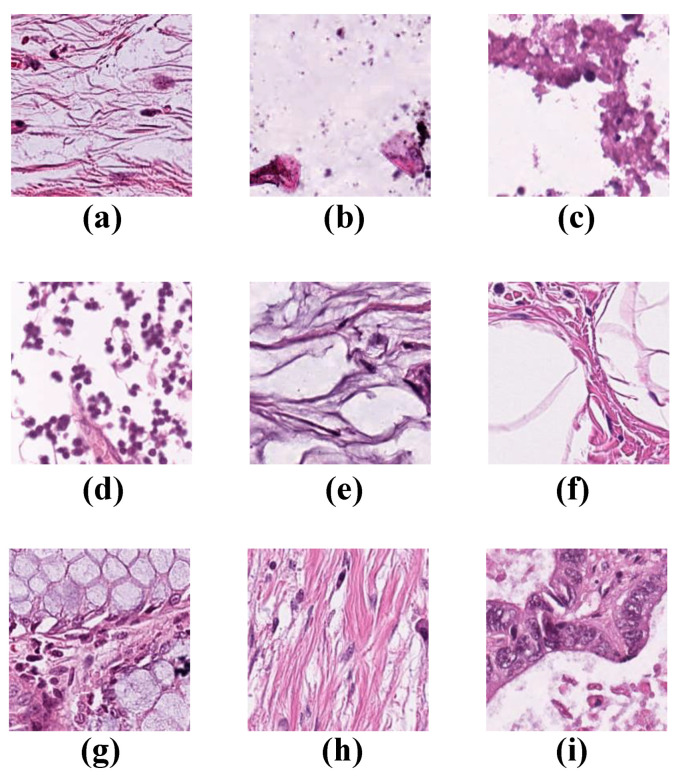
Example images for each of the nine tissue classes represented in the NCT-CRC-HE-100K datasets. (**a**) ADI: adipose tissue is composed mostly of adipocytes; (**b**) BACK: histological image background; (**c**) DEB: the debris is widely used in histopathology and diagnosis; (**d**) LYM: lymphocytes are the main type of cell found in the lymph; (**e**) MUC: mucus is produced by many tissues in the body and has a protective function; (**f**) MUS: smooth muscle; (**g**) NORM: tissues of the colon mucosa; (**h**) STR: stroma tissues of cancer-associated samples; (**i**) TUM: epithelium tissues of adenocarcinoma.

We used the open histology dataset of nine tissue classifications from NCT-CRC-HE-100K (data available at https://doi.org/10.5281/zenodo.1214456, 7 April 2018) to train the model in this experiment. The image dataset provided by Kather et al. [23] contains 86 hematoxylin and eosin-stained (H&E) slide tissues. Sample images of the nine tissue categories are shown in Figure 1. The data were derived from histological images of the available data on the NCT-UMM website, with all image dimensions 224 × 224 pixels (112 × 112 µm). Because the number of each type in the original dataset was different, we used the ratio of each type to obtain the corresponding number of training, validation, and tissue classification to ensure that the scale was satisfied. We split it into a 70% training dataset, 15% validation dataset, and 15% testing dataset, and presented the datasets to the model network for training, validation, and testing.

(2) Chest X-ray

**Figure 2:**
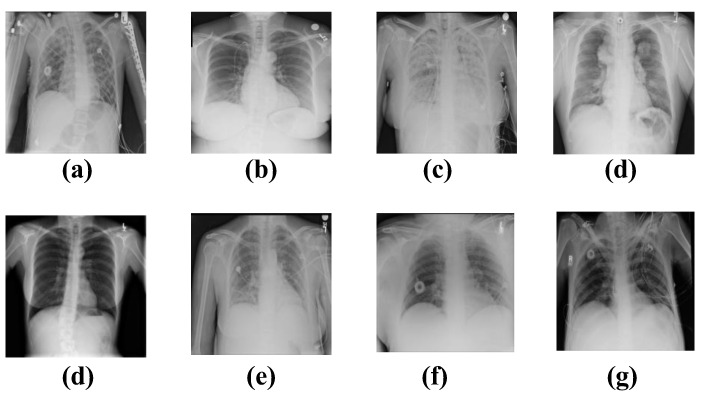
Eight common thoracic diseases observed in chest X-rays. (**a**) atelectasis, (**b**) cardiomegaly, (**c**) effusion, (**d**) infiltration, (**e**) mass, (**f**) Nodule, (**g**) pneumonia, and (**h**) pneumothorax.

We used the “ChestX-ray8” dataset (ChestX-ray8, data available at https://nihcc.app.box.com/v/ChestXray-NIHCC, accessed on 2 September 2017), which contained 108,948 images: 24,636 images that contained one or more pathologies and 84,312 images that were normal. The images in the ChestX-ray8 dataset were resized to 1024 × 1024 without a significant loss in detail. Eight common chest disease labels were included in the chest X-ray image, namely: (a) atelectasis, (b) cardiomegaly, (c) effusion, (d) infiltration, (e) mass, (f) nodule, (g) pneumonia, and (h) pneumothorax (Figure 2). In our experiments, we split the data into training (70%), validation (10%), and testing (20%) to identify eight chest diseases.

(3) Common skin lesions

Dermoscopy images are a standard source for training artificial neural networks to automatically diagnose pigmented skin lesions. We used the HAM10000 (“Human Against Machine with 10,000 training images”) dataset (HAM10000, data available at https://github.com/ptschandl/HAM10000_dataset, 4 June 2018), which consists of 10,015 dermoscopy images of different populations collected over 20 years from the Department of Dermatology of the Medical University of Vienna, Austria, and the Skin Cancer Clinic of Queensland, Australia. The dataset images are cropped from original images at 800 × 600 pixels. With the exception of mole-related melanoma, the image dataset was divided into seven general categories to exclude cases with indeterminate or ambiguous diagnosis classifications. Over 95% of all lesions encountered in clinical practice will fall into one of the following seven diagnostic categories;

(a) Apiece: actinic keratoses (solar keratoses) and intraepithelial carcinoma (Bowen’s disease) are common noninvasive variants of squamous cell carcinoma that can be treated locally without surgery.
(b) Bcc: basal cell carcinoma is a common variant of epithelial skin cancer that rarely metastasizes.
(c) Bkl: horny growth, especially on the skin, is a generic class that includes seborrheic keratosis and solar lentigo.
(d) Df: dermatofibroma is a benign skin lesion which is regarded as either benign proliferation or minimal trauma.
(e) NV: melanocytic nevi are benign neoplasms of melanocytes.
(f) Mel: melanoma is a malignant neoplasm derived from melanocytes that may appear in different variants.
(g) Vasc: vascular skin lesions.

(4) Diabetic retinopathy

A retinal OCT image is currently the most commonly used standard for retinal pathology, with approximately 30 million OCT scans performed globally each year [26]. Clear cross-sectional images of OCT can assist physicians in the early detection, diagnosis, and treatment of the main causes of blindness: age-related macular degeneration (AMD) and diabetic macular edema. The prevalence of these diseases is likely to increase further over time due to aging populations and the global diabetes epidemic. We passed an initial image quality review using Daniel [39] (OCT, data available at https://data.mendeley.com/datasets/rscbjbr9sj/2, accessed on 1 January 2018), providing 108,312 OCT images (37,206 choroidal neovascularization, 11,349 diabetic macular edemata, 8617 drusen, and 51,140 normal) from 4686 patients, which were divided into training (70%), validation (10%) and testing (20%) sets to train and test our CNN model network architecture.

(5) Pediatric chest X-ray

According to the World Health Organization (WHO), pneumonia kills about 2 million children under the age of five (Chest X-Ray, data available at https://data.mendeley.com/datasets/rscbjbr9sj/2, accessed on 6 January 2018). Bacterial and viral pathogens are the two main causes in clinical cases of pneumonia [35], with bacterial pneumonia requiring antibiotic treatment and viral pneumonia requiring referral for treatment. Therefore, accurate and timely use of chest X-ray image data is very important to distinguish different types of pneumonia. To this end, we used Daniel [39] to collect and label 5232 pediatric chest X-ray images from 5856 patients, 3883 of which were described as pneumonia (2538 bacteria and 1345 viruses) and 1349 normal, to train the model architecture. We tested our model using 234 normal and 390 pneumonia images (242 bacterial and 148 viral) from 624 patients.

(6) Breast ultrasound image

Breast cancer is one of the most common causes of mortality in women worldwide, but early detection can help to reduce it. The ultrasound breast cancer medical images provided by Walid [33] were used in this study (BUSI, data available at https://data.mendeley.com/datasets/rscbjbr9sj/2, accessed on 6 January 2018). The dataset consisted of 600 female patients aged 25 to 75 years, with 780 preprocessed breast ultrasound images in a PNG format. The average image size was 500 × 500 pixels and they were divided into three categories: normal (133 images), benign (487 images), and malignant (210 images). The data were divided into training (70%), validation (10%), and testing (20%) sets to train and test our CNN model network architecture in order to verify that breast ultrasound images can be used to detect and classify breast cancer. This produced good results in segmentation.

#### 3.1.3. Model Testing

To evaluate the performance of these CNN model network architectures, we firstly compared two methods of training network optimizers. Stochastic gradient descent momentum (SGDM) is an optimizer that normalizes gradients using the magnitude of recent gradients, while adaptive moment estimation (Adam) is an optimization algorithm that can be used for classical stochastic gradient descent. We then further compared the replacement mini-batch size and epoch to test the model.

### 3.2. Software and Tools Platform

In this study, the experiments were performed on a quad-core Intel(R) CPU i7-11700K@3.60GHz processor server with NVIDIA GeForce RTX 3090 GPU and OS Windows 10 system. MATLAB version R2021a software was adopted, which provides the pretrained deep neural network models including AlexNet, ResNet 50, Inception V3, DenseNet, MobileNet, and XceptionNet. Through the experiments, the training processes of the tested models could be visually demonstrated with accumulated accuracy and loss evaluation.

## 4. Experimental Results

Six common convolutional neural networks (CNN) models, AlexNet, ResNet, Inception V3, DenseNet, MobileNet, and XceptionNet, were used in this study. Firstly, we replaced the classification layer and used stochastic gradient descent momentum (SGDM) with adaptive moment estimation (Adam) optimizers to train the network. We then used our trained models to evaluate the performance of these network architectures on six different kinds of open datasets of medical images. Next, we compared them with the original paper’s experimental results to identify the model architecture that could best identify images of different disease tissue types and collated the experimental results from different datasets.

### 4.1. Colorectal Cancer Tissue

**Figure 3:**
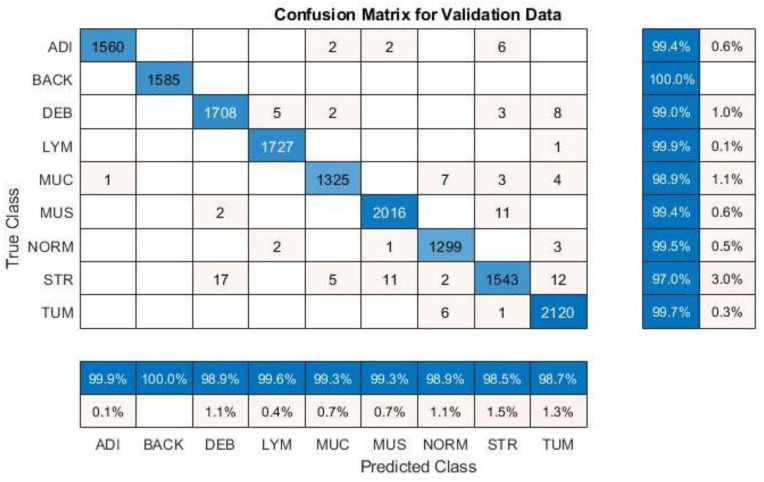
The accuracy of nine classes (CRC-VAL-HE-100K) with InceptionV3.

The data “NCT-CRC-HE-100K” of histological images for tissue training were used first, including 100,000 images of nine different tissue categories. The accuracy of each category is displayed by using a confusion matrix, as shown in Figure 3, and our experimental results are organized in Table 3. When no further improvement in accuracy was found after 150 epochs (iterations over the entire dataset), the training was stopped. In terms of the accuracy of multiple models, the highest accuracy of 99.43% was achieved with the Inception V3 model when using the adaptive moment estimation (Adam) optimizer to train the network. The best result using the stochastic gradient descent momentum (SGDM) optimizer was 99.19%, higher than the 98.7% accuracy obtained by Kather [23] in the original paper using VGG19.

### 4.2. Chest X-ray

**Figure 4:**
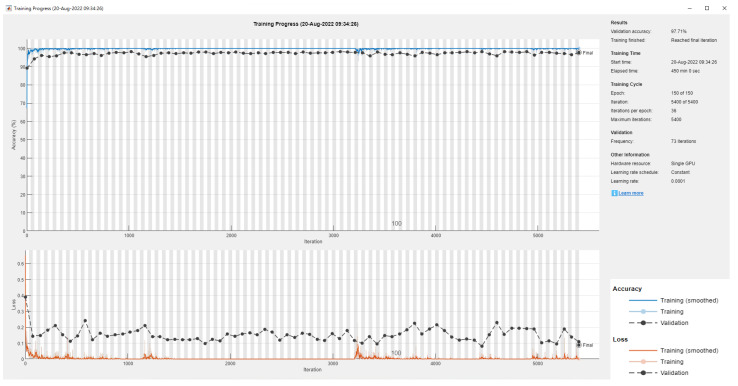
The accuracy of eight classes in ChestX-ray8 by InceptionV3.

The results of the experiments validated the multi-label classification ROC curves of eight chest disease categories using pretrained models with six CNN architectures, AlexNet, ResNet, Inception V3, DenseNet, MobileNet, and XceptionNet. We used the original image size (1024 × 1024) and increased the iteration size to accumulate gradients for more iterations. For the above CNN model, after using the adaptive moment estimation (Adam) optimizer to train the network, we set the batch size of the total number of training iterations to 128, and set the epoch to 100 to prevent overfitting and achieve convergence. The result obtained based on Inception V3 was 77.31%, as shown in Figure 4, which was higher than the 69.67% accuracy obtained in the original paper by Wang [25] using ResNet-50, and the best stochastic gradient descent momentum (SGDM) optimizer was used. The best result was 77.26%, and the experimental results are summarized in Table 4.

### 4.3. Common Skin Lesions

**Figure 5:**
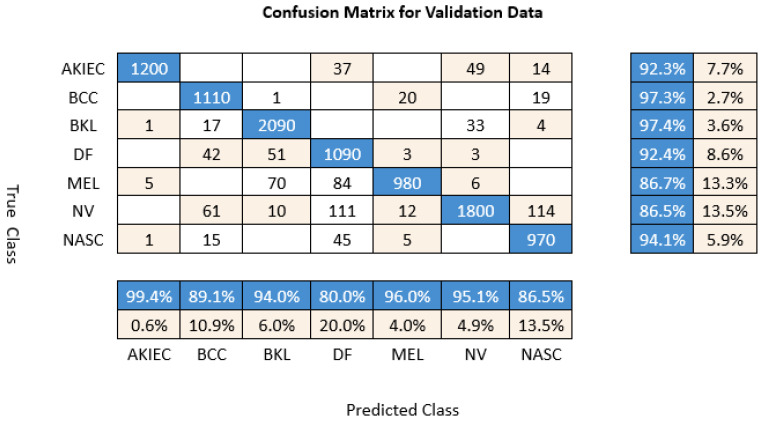
The experimental statistics of seven classes using HAM10000 data by InceptionV3.

**Figure 6:**
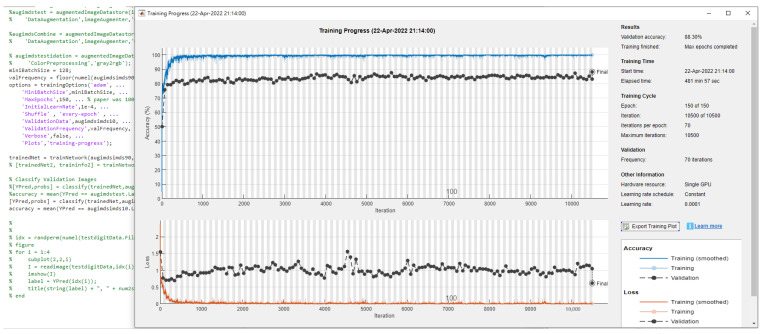
The accuracy of seven classes in HAM10000 by InceptionV3.

We fine-tuned the Inception V3 architecture (weights pretrained on ImageNet4 data) to classify images based on image type. After training the network using the adaptive moment estimation (Adam) optimizer for 150 epochs with a learning rate initialized to 0.0001 and a batch size of 128, the accuracy was sufficient to accelerate the classification process of the test dermoscopy images. We showed the experimental statistics of seven classes using HAM10000 data by InceptionV3 in Figure 5. The confusion matrix visualizes and summarizes the performance of the proposed classification algorithm. From Figure 5, the table categorizes the predictions against the actual values and the statistics demonstrate that the results are with high accuracy. Among these, the model based on Inception V3 achieved the best result of 88.3%, as shown in Figure 6. The experimental results are collated in Table 5. The best result using the stochastic gradient descent momentum (SGDM) optimizer was 86.00%, which was slightly lower than the 95% accuracy obtained in the original paper by Tschandl [26] using Inception V3. Even this study cannot yet repeat the high accuracy value of [26], and the authors still continue further to perform investigation to match the result. Currently, Inception V3 still claims the best performance during the literature review.

### 4.4. Diabetic Retinopathy

**Figure 7:**
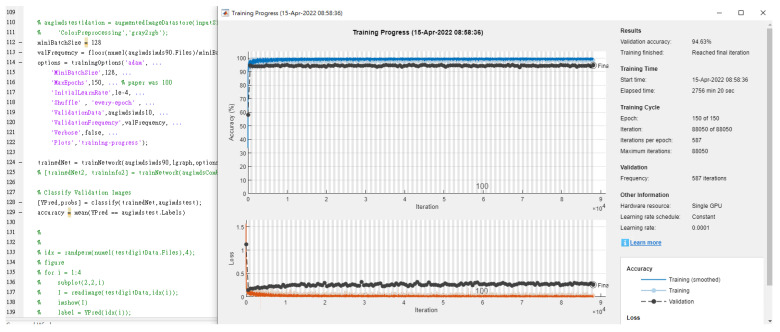
The accuracy of four classes in OCT by InceptionV3.

The retinal tomography (OCT) image classification was validated using a pretrained model with a CNN architecture. After using the adaptive moment estimation (Adam) optimizer to train the network, the entire dataset was iterated in the experiment. After 150 epochs, the learning rate was initialized to 0.0001 and the batch size was 64. As the accuracy had not further improved, the training was stopped. The model based on Inception V3 achieved the best result of 96.63%, as shown in Figure 7. The best result using the stochastic gradient descent momentum (SGDM) optimizer was 95.35%, which was higher than the 96.6% accuracy obtained in the original paper by Daniel [39] using Inception V3. The experimental results are summarized in Table 6.

### 4.5. Pediatric Chest X-ray

**Figure 8:**
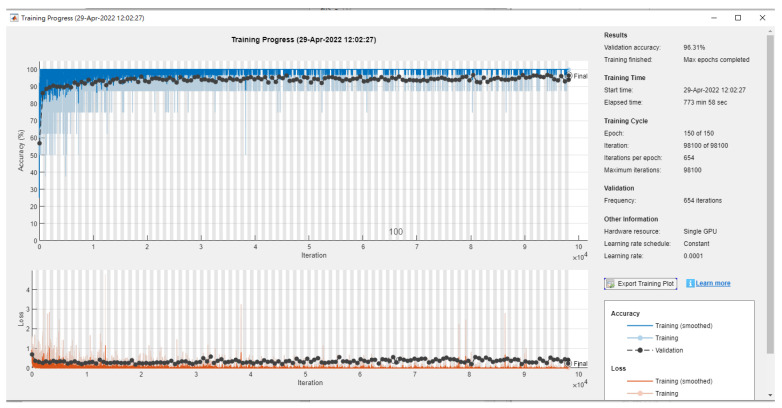
The accuracy of three classes in X-rays by InceptionV3.

We applied the transfer learning framework used to diagnose pediatric pneumonia to investigate the generalizability of six different CNN architecture pretrained models to diagnose common diseases. In comparing chest X-rays showing a normal level of pneumonia, we found that using the Inception V3-based model with the stochastic gradient descent momentum (SGDM) optimizer achieved an accuracy of 96.31%. This was higher than the 92.8% accuracy obtained in the original paper by Daniel [39] using Inception V3, and the best result using the adaptive moment estimation (Adam) optimizer was 96.27%. After the model iterated across the entire dataset for 100 epochs, the learning rate was initialized to 0.0001, and the batch size was eight. The training was stopped due to no further improvement in loss and accuracy (Figure 8). The experimental results are summarized in Table 7.

### 4.6. Breast Ultrasound Image

**Figure 9:**
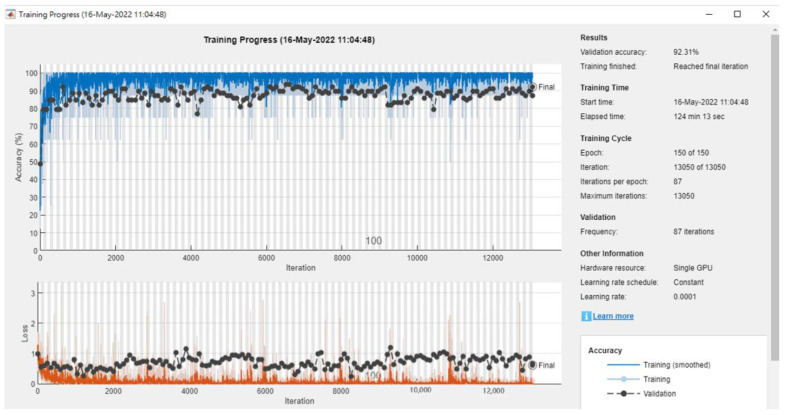
The accuracy of three classes in Breast by InceptionV3.

We conducted experiments using six different CNN models. We used a breast ultrasound dataset of 780 images classified into three categories: normal, benign, and malignant. After the adaptive moment estimation (Adam) optimizer was used to train the network, the model iterated the entire dataset for 150 epochs. The learning rate was initialized to 0.0001. The training was stopped after batch size eight, as there was no further improvement in loss and accuracy (Figure 9). Because Walid [33] did not mention the experimental results in the original paper, this paper only includes our experimental results. Based on the Inception V3 model, we achieved an accuracy of 92.31%, and the best result using the stochastic gradient descent momentum (SGDM) optimizer was 92.17%. These results are summarized in Table 8.

### 4.7. Data Analysis Section

After conducting experiments on six publicly available databases of pathological images, including colorectal cancer tissue, chest X-rays, common skin lesions, diabetic retinopathy, pediatric chest X-ray, and breast ultrasound image datasets. The accuracy alone is not enough to evaluate the performance of a model. We further compared the other measures such as sensitivity, specificity, F1 score, balanced accuracy in Table 9. From the data in Table 9, the experimental results demonstrate that our study can achieve high accuracy and consistent superior performance for different medical image datasets.

## 5. Conclusions

In this paper, we used a series of experiments, parameter optimization, and different deep learning parameter corrections to find a deep learning model that could produce the best classification accuracy in recognizing images of different tissues. Different CNN-based deep learning models were used to classify medical images. Six different histology images were used as experimental datasets, and the six most commonly used deep learning network models were compared to accurately identify the model that could best distinguish tissue image classification. The Inception V3 model was found to have a high recognition rate and outperformed other network models in the experimental results. In short, Inception V3 outperformed the techniques described in the literature. It achieved the best classification accuracy and was tested on a ChestX-ray8 [28] dataset of chest X-ray images, reaching 77.31%, which was far more than using 69.67% of the original paper for ResNet50. On the breast ultrasound image dataset, Inception V3 achieved an accuracy of 92.31%, exceeding the 81.88% accuracy of the second-highest model, Resnet50. Therefore, this successfully demonstrates the wide applicability of the Inception V3 model in classifying different medical images, which can help physicians to enhance their critical thinking skills and make the most appropriate decisions during the diagnostic process.

We used different CNN deep neural networks to obtain the best experimental results. However, in many fields of clinical medicine, the technology of deep CNN still has two main obstacles: large amounts of data and well-annotated datasets are required, and these datasets are difficult to obtain and annotate. Additionally, in a wide range of clinically relevant situations, there are a lack of proposed results using the trained CNN model for comparison and verification. Our research can serve as an objective recommendation for clinical evaluation, and our proven best model approach can also be used to automatically detect many more different histological image classifications and applications. Finally, Table 10 summarizes the difference between our research and other existing works for six medical image datasets.

## Figures and Tables

**Table 1:** Research resources.

Literature	Research Objective	Classification Technique	The Best Classification Technique	Accuracy Rate (%)
[14]	Predicting survival from colorectal cancer histology slides using deep learning, a retrospective multicenter study	VGG19, AlexNet, SqueezeNet, GoogLeNet, Resnet50	VGG19	98.7
[20]	ChestX-ray8 hospital-scale chest X-ray database and benchmarks on weakly supervised classification and localization of common thorax diseases	AlexNet, GoogLeNet, VGGNet-16, ResNet-50	ResNet-50	69.67
[23]	The HAM10000 dataset, a large collection of multi-source dermatoscopic images of common pigmented skin lesions	Inception V3	Inception V3	95
[9]	Identifying medical diagnoses and treatable diseases with image-based deep learning	Inception V3 for Octmnist	Inception V3	96.6
[9]	Identifying medical diagnoses and treatable diseases with image-based deep learning	Inception V3 for Pneumoniamnist	Inception V3	92.8
[24]	Dataset of breast ultrasound images	-	-	-

**Table 2:** Histological image dataset.

Dataset	Image Number	Training	Validation	Testing	Number of Classes	Image Size
NCT-CRC-HE-100K [14]	107,180	89,996	10,004	7180	9	224 × 224
ChestX-ray8 [20]	112,120	78,468	11,219	22,433	8	512 × 512
Human Against Machine with 10,000 training images [23]	10,015	7007	1003	2005	7	600 × 450
Optical coherence tomography (OCT) images [9]	109,309	97,477	10,832	1000	4	512 × 496
Chest X-Ray Images [9]	5856	4708	524	624	2	944 × 940
Breast ultrasound images [24]	780	546	78	156	3	562 × 471

**Table 3:** The best result of NCT-CRC-HE-100K.

Model	Accuracy Rate% (Times)	
	Adam	SGDM
AlexNet	68.86% (395 min)	67.48% (412 min)
ResNet 50	99.39% (720 min)	99.01% (733 min)
ResNet 18	99.37% (422 min)	99.15% (458 min)
Inception V3	**99.43% (2658 min)**	99.19% (2683 min)
DenseNet	81.25% (1964 min)	81.07% (1990 min)
MobileNet	80.51% (508 min)	80.25% (533 min)
XceptionNet	81.49% (2643 min)	81.04% (2697 min)

Bold symbols represent the maximum values of each column in the tables.

**Table 4:** The best result of Chest X-ray8.

Model	Accuracy Rate% (Times)	
	Adam	SGDM
AlexNet	76.99% (177 min)	76.91% (182 min)
ResNet 50	77.28% (447 min)	77.24% (430 min)
ResNet 18	77.89% (194 min)	77.84% (188 min)
Inception V3	**77.31% (1256 min)**	77.26% (1283 min)
DenseNet	75.18% (2025 min)	75.17% (2034 min)
MobileNet	75.25% (2018 min)	75.17% (2034 min)
XceptionNet	75.37% (2017 min)	75.17% (2034 min)

Bold symbols represent the maximum values of each column in the tables.

**Table 5:** The best result of Human Against Machine with 10,000 training images.

Model	Accuracy Rate% (Times)	
	Adam	SGDM
AlexNet	83.42% (45 min)	83.37% (53 min)
ResNet 50	86.6% (170 min)	86.42% (185 min)
ResNet 18	86.6% (170 min)	86.39% (193 min)
Inception V3	**88.3% (481 min)**	86.00% (497 min)
DenseNet	88.19% (473 min)	88.24% (495 min)
MobileNet	88.02% (468min)	88.13% (490 min)
XceptionNet	88.17% (502min)	88.21% (499 min)

Bold symbols represent the maximum values of each column in the tables.

**Table 6:** The best result of Optical coherence tomography (OCT) images.

Model	Accuracy Rate% (Times)	
	Adam	SGDM
AlexNet	93.11% (2651 min)	92.07% (2666 min)
ResNet 50	93.89% (1506 min)	93.68% (1532 min)
ResNet 18	94.24% (1831 min)	94.09% (1857 min)
Inception V3	**96.63% (3383 min)**	95.35% (3392 min)
DenseNet	68.12% (2473 min)	68.04% (2485 min)
MobileNet	68.31% (973 min)	68.18% (996 min)
XceptionNet	68.69% (2595 min)	68.26% (2657 min)

Bold symbols represent the maximum values of each column in the tables.

**Table 7:** The best result of Chest X-Ray Images.

Model	Accuracy Rate% (Times)	
	Adam	SGDM
AlexNet	85.29% (104 min)	85.22% (114 min)
ResNet 50	92.66% (141 min)	92.47% (167 min)
ResNet 18	90.84% (98 min)	90.64% (103 min)
Inception V3	96.27% (762 min)	**96.31% (774 min)**
DenseNet	90.21% (251 min)	90.20% (257 min)
MobileNet	88.12% (45 min)	88.03% (49 min)
XceptionNet	89.94% (130 min)	89.77% (145 min)

Bold symbols represent the maximum values of each column in the tables.

**Table 8:** The best result of Breast ultrasound images.

Model	Accuracy Rate% (Times)	
	Adam	SGDM
AlexNet	75.73% (12 min)	75.65% (14 min)
ResNet 50	81.88% (18 min)	81.67% (21 min)
ResNet 18	72.73% (14 min)	72.59% (17 min)
Inception V3	**92.31% (29 min)**	92.17% (33 min)
DenseNet	70.91% (27 min)	70.86% (30 min)
MobileNet	61.86% (6 min)	61.72% (7 min)
XceptionNet	72.11% (15 min)	72.03% (17 min)

Bold symbols represent the maximum values of each column in the tables.

**Table 9:** The sensitivity, specificity, F1 score, balanced accuracy of six dataset.

Dataset	Sensitivity	Specificity	F1 Score	Balanced Accuracy (%)
NCT-CRC-HE-100K	0.99	0.99682	1.8965	99.42
ChestX-ray8	0.76	0.64192	1.0143	76.98
Human Against Machine with 10,000 training images	0.97368	0.83333	1.3528	87.9
Optical coherence tomography (OCT) images	0.96	0.96225	1.3018	96.62
Chest X-Ray images	0.96	0.96891	1.2541	96.34
Breast ultrasound images	0.95	0.95556	1.1339	93.59

**Table 10:** The difference between our research and other existing works in this area.

	Existing Researches			Our Research		
Dataset	Classification Technique	The Best Classification Technique	Accuracy Rate (%)	Classification Technique	The Best Classification Technique	Accuracy Rate (%)
NCT-CRC-HE-100K [14]	VGG19, AlexNet, SqueezeNet, GoogLeNet, Resnet50	VGG19	98.7	AlexNet, ResNet 50, ResNet 18, Inception V3, DenseNet, MobileNet, XceptionNet	Inception V3	99.43
ChestX-ray8 [20]	AlexNet, GoogLeNet, VGGNet-16, ResNet-50	ResNet-50	69.67		Inception V3	77.31
Human Against Machine with 10000 training images [23]	Inception V3	Inception V3	95		Inception V3	88.3
Optical coherence tomography (OCT) images [9]	Inception V3	Inception V3	96.6		Inception V3	96.63
Chest X-Ray Images [9]	Inception V3	Inception V3	92.8		Inception V3	96.31
Breast ultrasound images [24]	-	-	-		Inception V3	92.31

## Data Availability

All data are publicly available.
